# Lactoferrin quantification in cattle faeces by ELISA

**DOI:** 10.7717/peerj.8631

**Published:** 2020-02-27

**Authors:** Andrew S. Cooke, Kathryn A. Watt, Greg F. Albery, Eric R. Morgan, Jennifer A.J. Dungait

**Affiliations:** 1 Rothamsted Research, North Wyke, Okehampton, UK; 2 School of Veterinary Science, University of Bristol, Bristol, UK; 3 Institute of Evolutionary Biology, University of Edinburgh, Edinburgh, UK; 4 Institute of Global Food Security, The Queen’s University Belfast, Belfast, UK; 5 College of Life and Environmental Sciences, University of Exeter, Exeter, UK

**Keywords:** One-health, Livestock, Veterinary science, Animals, Immunology, Ecology, Agriculture, Veterinary medicine, Zoology

## Abstract

**Background:**

Promoting and maintaining health is critical to ruminant welfare and productivity. Within human medicine, faecal lactoferrin is quantified for routine assessment of various gastrointestinal illnesses avoiding the need for blood sampling. This approach might also be adapted and applied for non-invasive health assessments in animals.

**Methods:**

In this proof-of-concept study, a bovine lactoferrin enzyme-linked immunosorbent assays (ELISA), designed for serum and milk, was applied to a faecal supernatant to assess its potential for quantifying lactoferrin in the faeces of cattle. Faecal lactoferrin concentrations were compared to background levels to assess the viability of the technique. A comparison was then made against serum lactoferrin levels to determine if they were or were not reflective of one another.

**Results:**

The optical densities of faecal samples were significantly greater than background readings, supporting the hypothesis that the assay was effective in quantifying faecal lactoferrin (*T*_13, 115_ = 11.99, *p* < 0.0005). The mean faecal lactoferrin concentration was 0.269 µg mL^−1^ (S.E. 0.031) and the mean serum concentration 0.074 µg mL^−1^ (S.E. 0.005). Lactoferrin concentrations of faecal and serum samples, taken from the same animals on the same day, were significantly different (*T*_21_ = 2.20, *p* = 0.039) and did not correlate (*r* = 0.2699, *p* = 0.238).

**Conclusion:**

Results support the hypothesis that lactoferrin can be quantified in cattle faeces by ELISA. Whilst further research is required to determine the physiological source of the lactoferrin, this highlights the potential of the method for non-invasive assessment of cattle immunology and pathology.

## Introduction

Ruminant health is central to ensuring animal welfare and to facilitating productivity and sustainability in commercial ruminant systems. Inflammation, particularly at the intestinal barrier, can be symptomatic of poor health and cause significant welfare and economic losses through reduced feed conversion and productivity (Beever & Doyle, 2007; Charlier et al., 2009; Khiaosa-ard & Zebeli, 2014; Niezen et al., 1995; VandeHaar & St-Pierre, 2006). Kvidera et al. (2017) found that a compromised intestinal barrier, causing inflammation, significantly reduced feed intake and milk yields in dairy cattle, in beef cattle this would likely result in a reduction in weight gain, with an economic cost. As worldwide demand for meat increases (Fiala, 2008), along with pressures on the natural resources that support its production (Gordon et al., 2017), it is essential that gut health is optimised to improve the efficiency and sustainability of livestock production systems. This calls for the urgent development of economically viable tools for the rapid diagnosis of gut disease, to support prevention and rapid correction of poor gut function.

Recently, Watt et al. (2016) and Cooke et al. (2019) both demonstrated that enzyme-linked immunosorbent assays (ELISA), designed for use on serum and milk, can be utilised for quantifying anti-parasite antibodies in the faeces of sheep and cattle, through the use of a novel faecal supernatant (Watt et al., 2016; Cooke et al., 2019). This suggests the possibility of quantifying other immunological proteins in faeces. Such techniques can provide valuable insights into the health of livestock, particularly in relation to parasitic diseases and potentially gastrointestinal health in general. Another advantage of faeces-based methods is that samples can be collected non-invasively and without negative impacts on welfare. Wider potential benefits include the immunological assessment of animals that cannot be directly sampled, for example, if they are evasive or dangerous.

Lactoferrin is an inflammatory marker and key indicator of gut damage. Lactoferrin binds to iron, preventing its utilisation by bacteria and producing a bacteriostatic effect (Weinberg, 1984). For example, Kieckens et al. (2015) found that rectal administration of lactoferrin to calves significantly reduced *Escherichia coli* counts. Furthermore, lactoferrin can regulate immune responses against infection, preventing inflammation by modulating immune cell function, migration and maturation (Kruzel, Zimecki & Actor, 2017; Legrand et al., 2005). Although predominantly found at mucosal surfaces, lactoferrin can be detected in milk and serum (Sánchez, Calvo & Brock, 1992). In human medicine, faecal lactoferrin is used as an inflammatory marker in the diagnosis of gastrointestinal conditions such as inflammatory bowel diseases and Crohn’s disease (Gisbert et al., 2009; Lamb & Mansfield, 2011; Lundberg et al., 2005; Tibble et al., 2000). This is due to the localised expression of lactoferrin throughout the body. One key site of lactoferrin expression is by epithelial cells for secretion to mucosal surfaces (Ward, Paz & Conneely, 2005), such as those lining the intestinal tracts of mammals. However, lactoferrin is also present in other parts of the body such as hepatocytes (Oh et al., 1997) and blood cells (Bennett & Kokocinski, 1978). Despite lactoferrin analysis being common place in human medicine, quantification of lactoferrin is not routinely conducted within veterinary medicine, other than for the analysis of bulk-tank milk (Nielsen et al., 2000; Parker et al., 2017; Stabel, Wells & Wagner, 2002; Thobokwe, Heuer & Hayes, 2004).

The purpose of this study is to evaluate the feasibility of quantifying lactoferrin in ruminant faeces using techniques analogous to those presented by Watt et al. (2016) and Cooke et al. (2019). That is, to assess if lactoferrin can be quantified in the faeces of ruminants by using a commercially feasible ELISA product, designed for serum and milk, with a specialised faecal supernatant.

## Methods

### Sample populations

Faecal samples were collected from three herds of beef cattle located in Cornwall, Angus, and Hertfordshire in the UK (C1, C2 and C3 respectively). Across the groups, cattle included calves, cows, heifers and steers. Groups C1 and C3 were fed on grass silage for at least 1 month prior to sampling and group C2 was permanently grazed on pasture. Group C1 were Aberdeen Angus crossed with Belgian Blue and Holstein. Group C2 were all Aberdeen Angus crossed with Hereford and Galloway. Group C3 were the Sussex breed. A total of 117 faecal samples were collected from the three farms (65, 30 and 22 from C1, C2 and C3 respectively), and 22 blood samples were collected from C3. Sample numbers were based on availability, gathering the most feasible from each farm. Cattle had no known or visually obvious signs of illness or infirmity at the time of sampling, however individual animal health differences were not a focus of the study and clinical health assessments were not conducted.

Access to study sites was permitted by the landowner/manager. Permissions for the cattle sites were obtained by Andrew Cooke (Rothamsted Research) and permissions for the sheep and deer sites were obtained by the University of Edinburgh.

All cattle blood samples were taken by a trained and qualified veterinary surgeon who was conducting routine analysis in support of animal health under the UK Veterinary Surgeons Act 1966. Samples were analysed on request of the veterinary surgeon using excess samples and did not require excess blood being drawn in addition to what wasrequired under routine practice. Sheep blood samples were taken under the Animal Scientific Procedures Act (1986). Samples used in this study were remnant samples taken under project license no: PPL 60/4211, personal license no: PIL 60/623. The single deer blood sample was taken with the landowner’s permission from an animal shot for food.

Some sheep and deer samples (faeces and blood) were available from other experiments and were analysed as an additional data set. No commercially feasible lactoferrin ELISA was available for analysis of those samples, so they were subject to the bovine lactoferrin ELISA protocol. Further details of the analysis of sheep and deer samples are available in the Supplemental Material.

### Sample collection and preparation

#### Faecal samples

Fresh faeces were collected from the ground immediately after defecation was observed. Faecal samples were stored in screw-top 100 mL plastic containers and stored at −18 °C until processing. Samples were defrosted at room temperature and mixed with a protease inhibitor (cOmplete™, EDTA-free Protease Inhibitor Cocktail, Roche, Germany) at a ratio of 1:1–1:2 (w:v) with phosphate-buffered saline, as per the manufacturers protocol, depending upon consistency and moisture. This mixture was homogenised and centrifuged at 3–6 °C and 12,000×*g* (Sorvall SLA-3000 rotor in a Sorvall RC-5B centrifuge, ThermoFisher Scientific, Waltham, MA, USA) for 5 min. The faecal supernatant was then removed by pipette and stored at −18 °C.

#### Blood samples

Blood samples were collected by tail venipuncture into glass Vacutainers^®^ (Becton Dickinson, Franklin Lakes, NJ, USA). Samples were left >30 min to clot and were centrifuged at 1,056×*g* (Sorvall SLA-3000 rotor in a Sorvall RC-5B centrifuge) for 15 min to separate serum, which was removed by pipette and stored at −18 °C.

#### Serial dilutions

Test plates were conducted to determine the optimum concentration of faecal supernatants and serum (diluted with Tris-buffer saline with 0.05% Tween™ 20 (TBST)) to achieve optical densities within the detection limits of the plate reader (Spectramax M2) and to best show the variation in the datasets. The optimum dilution was qualitatively determined as the dilution at which no notable plateauing or data clumping had begun, which can both be features of more dilute samples. If two dilutions presented similar qualities, the least concentrated was chosen to ensure the capture of lactoferrin samples higher than those on the trial plates and to preserve sample quantity. Test plates were conducted on 63 cattle faecal samples at concentrations of 1/1, 1/2, 1/8 and 1/32 and on 22 cattle serum samples at concentrations of 1/5, 1/10, 1/20 and 1/40.

On each plate, a seven-point halving series dilution of bovine lactoferrin standard was included (Bethyl Laboratories; RC10-126-8) for reference and as a positive control (derived from bovine colostrum (L4765; Merck, Darmstadt, Germany)). Stock solution was 1,000 µg mL^−1^, and for the first standard in the series was diluted to 0.5 µg mL^−1^ with TBST. Subsequent dilutions added 500 µL of the previous solution in the series to 500 µL of TBST. Each plate also included three negative control blanks of TBST.

### ELISA protocol

The ELISA was conducted using a commercially available bovine lactoferrin ELISA set (E10-126B; Bethyl Laboratories Inc., Montgomery, TX, USA) which is produced primarily for use on bovine milk samples. The specified limit of detection for the product is 7.8–500 ng ml^−1^. The manufacturers protocol was followed as closely as possible, including the use of the specified buffers and reagents.

The plate coat was made by mixing affinity purified antibody (A10-126A; Bethly Laboratories Inc., Montgomery, TX, USA) with carbonate buffer at a ratio of 1:100 (v:v). Then, 100 µL of the formed coat was added to each well and the plates (Nunc–Immuno Maxsorp 96-well) were covered in cling film and incubated at 20 °C for 1 h.

After the first incubation, the plates were washed five times with TBST using an automated plate washer (Skan Washer 400). A total of 200 µL of TBST was added to each well as a blocking solution and plates were covered in cling film and incubated at 20 °C for 30 min.

After the second incubation, the plates were washed five times in TBST before 100 µL of sample was added to each well (except blanks) and plates were covered in cling film and incubated at 20 °C for 1 h.

After the third incubation, the plates were washed five times in TBST before 100 µL of horseradish peroxidase (HRP) detection (0.5% with carbonate buffer) antibody was added to each well and the plates were covered in cling film and incubated at 20 °C for 1 h.

After the fourth incubation, the plates were washed five times in TBST before 100 µL of enzyme-substrate (SureBlue™ TMB Microwell Peroxidase Substrate Kit, SeraCare, Milford, MA, USA) was added to each well before the plates were placed in opaque boxes and incubated at 20 °C for 15 min. Then, 100 µL of stop solution, 0.18 m H_2_SO_4_, was added to each well and plates were immediately read for optical density at 450 nm by a plate reader, detecting the interaction of the enzyme and substrate.

### Statistical analysis

All statistical analyses were performed to a confidence level of 95% in Minitab 18 (Minitab Ltd., Coventry, UK). Two-sample *T*-tests were used to determine if faecal supernatant ODs were significantly above background levels (TBST blanks). Pearson’s correlations were performed to determine if the test results for serum and faecal samples, matched per individual and taken on the same day, were correlated. A Pearson’s correlation was also performed to assess if faecal sample moisture correlated to lactoferrin concentration.

The limit of detection (LoD) (Tholen, 2004; Armbruster & Pry, 2008) was calculated through the limit of blanks (LoB) using optical density values of blanks and reference material as:

$${\rm{LoB}} = {\rm{mea}}{{\rm{n}}_{{\rm{blanks}}}} + 1.645\;({\rm{SD}_{{\rm{blanks}}}})$$

$${\rm{LoD}} = {\rm{LoB}} + 1.645\;\left( {{\rm{S}}{{\rm{D}}_{{\rm{low}}\;{\rm{concentration}}\;{\rm{sample}}}}} \right)$$

The low concentration sample used was the lowest concentration serial dilution of reference material which was at a concentration of 0.007815 µg mL^−1^. The optical density generated was then converted to an estimated lactoferrin concentration by interpolating the LoD optical density onto the mean reference curve of all plates through a four-parameter sigmoidal curve.

## Results

### Controls, references and calibration

Negative controls of TBST were consistent across all plates and had a mean background optical density of 0.0486, ranging from 0.0467 to 0.0506, with a relative standard error of 0.77%. Reference material gave calibration consistent curves with a mean relative standard error of 1.51% across all dilutions.

**Figure 1:**
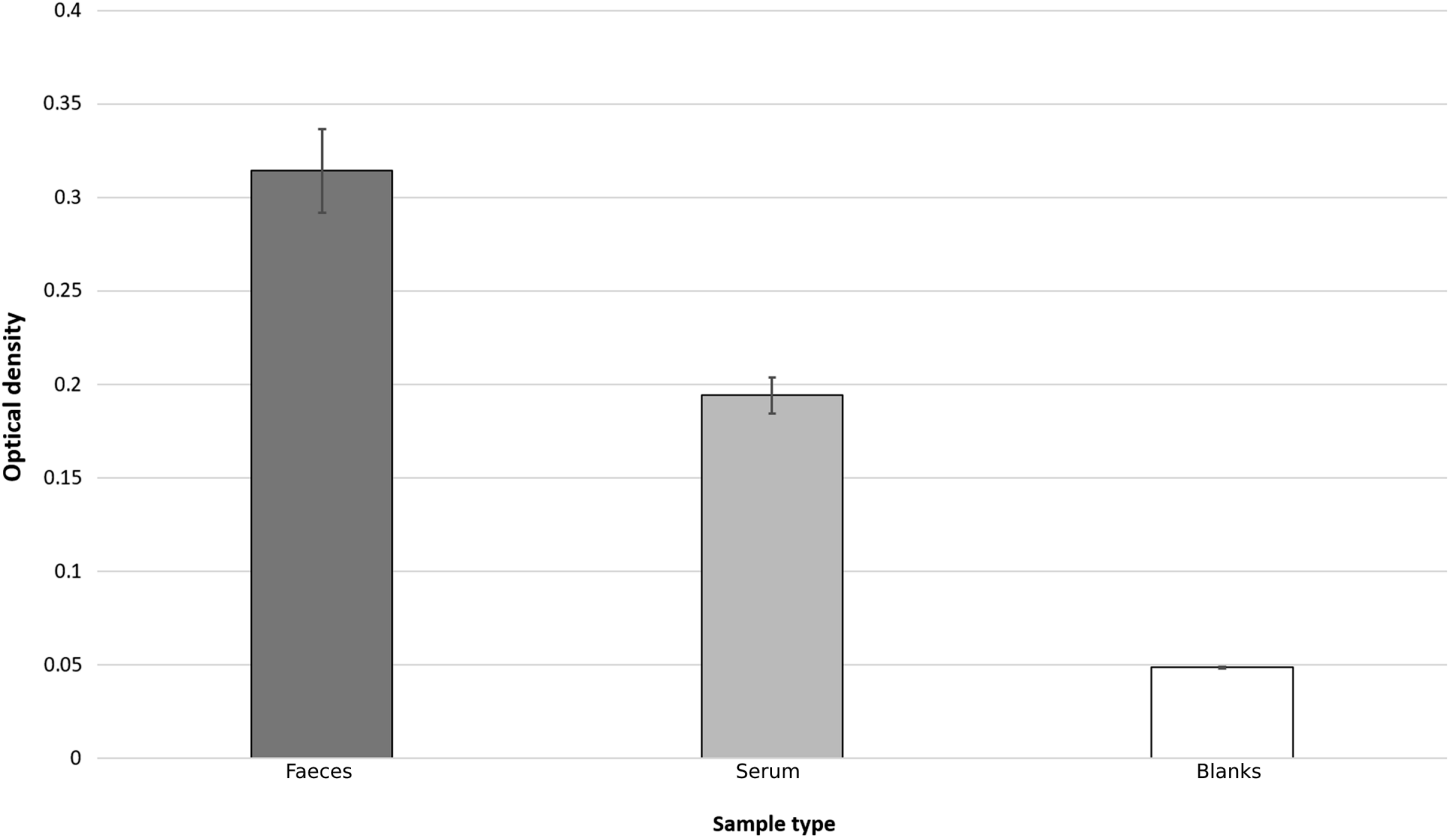
Bar chart showing average optical densities, after ELISA process, of faecal, serum and TBST blank samples. Error bars represent standard error.

Based on the results of three initial test plates, it was determined that a sample concentration of 50% (v:v with TBST) was optimum for cattle faecal samples. At this dilution, all samples yielded optical densities significantly above background levels, as determined using 2-sample *T*-tests that compared background levels to faecal supernatants (*T*_13, 115_ = 11.99, *p* < 0.0005) (Fig. 1).

### Lactoferrin concentrations

**Figure 2:**
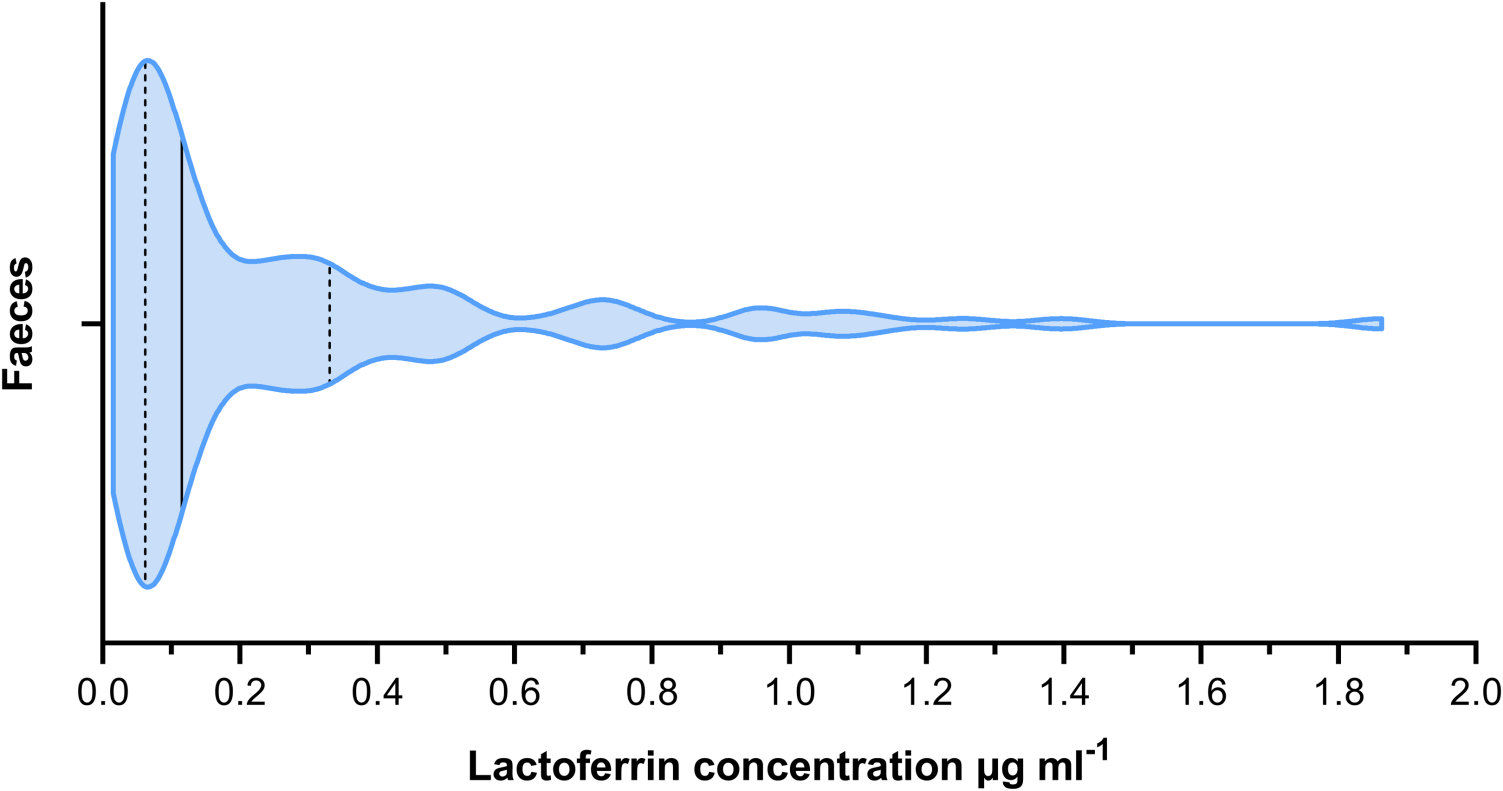
Violin plot showing the distribution of faecal lactoferrin concentrations (µg mL^−1^) of faecal samples from cattle (*n* = 115). The solid black line indicated the median value. The two dashed lines represent the 1st and 3rd quartiles respectively. The width of the plot at any given point represents the frequency of values in that region of the plot.

**Figure 3:**
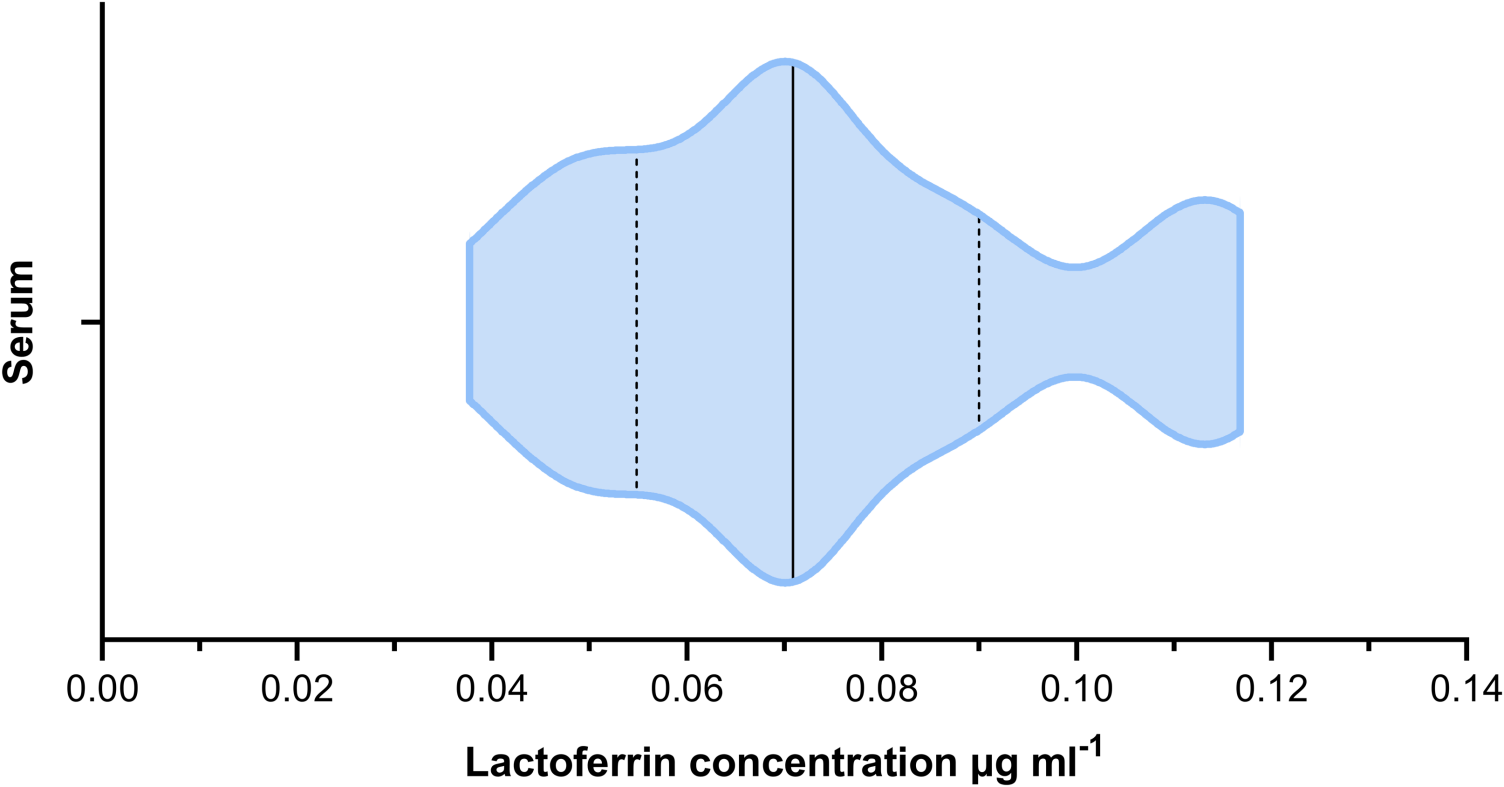
Violin plot showing the distribution of serum lactoferrin concentrations (µg mL^−1^) from cattle (*n* = 22). The solid black line indicated the median value. The two dashed lines represent the 1st and 3rd quartiles respectively. The width of the plot at any given point represents the frequency of values in that region of the plot.

The mean lactoferrin concentration across all faecal samples was 0.269 µg mL^−1^ (Fig. 2). The relative standard error was 0.031 and the coefficient of variance 1.236. For serum samples, the mean concentration was 0.074 µg mL^−1^ (Fig. 3). The relative standard error was 0.005 and the coefficient of variance was 0.314. A paired *T*-test comparing matched faecal and serum samples from the same individuals, taken on the same day, found a statistically significant difference between serum and faecal lactoferrin (*T*_21_ = 2.20, *p* = 0.039). Furthermore, no statistically significant correlation was found between faecal and serum lactoferrin concentrations taken from the same individuals on the same day (*r* = 0.269, *p* = 0.238).

The LoD of faecal samples generated an optical density of 0.0561, which was interpolated as a lactoferrin concentration of 0.0025 µg ml^−1^.

## Discussion

The optical densities of faecal and blood samples, isolated according to the extraction methods, significantly exceeded those from blank controls (TBST), showing active binding of the capture antibody during the assay. It is, therefore, concluded that the ELISA protocol was successful for the detection of lactoferrin in faeces of cattle through the use of a faecal supernatant. Sheep and deer samples taken in Scotland (see Supplemental Material) also provided optical densities significantly above background levels, however to a lower extent than the cattle faecal samples. Whilst this may be a genuine difference, it may be due to sub-optimal binding of non-bovine lactoferrin to a bovine-specific assay, further supporting the validity of the assay to specifically detect lactoferrin. We, therefore, accept the hypothesis that lactoferrin can be quantified in the faeces of ruminants using commercially available ELISA products. However, cross-species ELISAs are likely to have reduced avidity and therefore results between different species should not be compared if using the same ELISA, whilst also having a potentially reduced limit of detection.

The lack of correlation between faecal and serum lactoferrin, taken from the same individuals on the same day, suggests that faecal lactoferrin quantification is unlikely to be a suitable proxy for systemic lactoferrin. It may be the case that the faecal lactoferrin detected originated from a different localised physiological source to the serum lactoferrin. Therefore, their relative abundances would not be expected to correlate. This would indicate the potential for future research to determine what source faecal lactoferrin is representative of. One candidate is the mucosal lining of the gastrointestinal tract, which would mean that faecal lactoferrin may be an indicator of gastrointestinal health and pathology. Variation in localised lactoferrin expression has been previously described for lactoferrin production in the human body (Adlerova, Bartoskova & Faldyna, 2008; Levay & Viljoen, 1995), though literature is limited for ruminants. It is important to consider that the structure of lactoferrin varies throughout the body and that the assay used was designed for milk and serum and therefore the avidity of faecal lactoferrin, depending on its source, may be sub-optimal. However, assuming the source of faecal lactoferrin is different to that of milk and serum, this is not an issue so long as the assays avidity to faecal lactoferrin is consistent and that faecal lactoferrin data is only used comparatively to other faecal lactoferrin data. Confirmation of the gut as the physiological source of the lactoferrin in the faeces of ruminants could be achieved by taking swab samples for analysis along gastrointestinal transects of recently slaughtered individuals and comparison with matched faecal samples taken immediately prior to death. However, the contrasting chemistries of ruminant faeces and blood and the manner in which lactoferrin reacts to different organic and inorganic molecules may affect the successful extraction of the immune-marker from the different substrates. For example, inconsistent avidity of the ELISA to different physical forms of lactoferrin has been identified (Bagby & Bennett, 1982; Bennett, Bagby & Davis, 1981; Kanyshkova, Buneva & Nevinsky, 2001; Mantel, Miyazawa & Broxmeyer, 1994).

Based on the complexities related to the presentation of lactoferrin in faeces, future methodological development should include a comparison with complementary established techniques in molecular biology that detect specific proteins, for example, western blot and mass spectroscopy, to ratify the results obtained by ELISA to establish its reliability. However, lactoferrin ELISAs are established and recognised (Hetherington, Spitznagel & Quie, 1983), and widely used to quantify human lactoferrin in faeces (Gisbert et al., 2009; Lamb & Mansfield, 2011; Buderus et al., 2004) and bovine lactoferrin in milk and serum (Cheng et al., 2008), supporting the potential for the use of this technique for quantifying bovine faecal lactoferrin. The purpose of this study was to determine the feasibility of the technique and therefore detailed individual animal health measurements were not taken. Now that evidence for feasibility has been achieved, further advancement could be made through the sampling of cattle in a range of different health conditions, particularly those with known gastrointestinal pathogens/diseases such as coccidiosis and gastrointestinal helminth infections. This would provide information as to the practical use of the assay as a diagnostic tool.

This study highlights the need and opportunity for the development of testing methods to assess animal health using faeces, as opposed to blood and milk, as the testing material. Other biomarkers may be suitable for analogous work, particularly biomarkers such as calprotectin (Gisbert et al., 2009; Lamb & Mansfield, 2011; Lundberg et al., 2005) and haptoglobin (Kvidera et al., 2017; Quaye, 2008).

## Conclusion

The objective of this research was to assess the feasibility of quantifying lactoferrin in the faeces of cattle using a specialised faecal supernatant. This objective was met, and the assay deemed feasible. Whilst prior work using faecal supernatants has focused on antibodies, the results with lactoferrin results promote the utility of a faecal supernatant as a useful substrate for quantifying a wider range of biomolecules. This also opens the potential future development of using faecal lactoferrin as a health indicator in ruminants. However, this research is only a preliminary step towards that and achieving such would require significant further work to better understand the mechanisms involved.

The development of a rapid and non-invasive test for the gut health of ruminants using faecal lactoferrin quantification has potentially wide-reaching benefits. Immunological assessments of mammals are typically invasive and can be logistically difficult due to animal aggression, evasiveness and animal welfare legislation. Faecal sampling, therefore, offers an opportunity for the wide-ranging assessment of gut health in mammals.

This study trialled an existing ELISA, developed to quantify lactoferrin concentrations in milk, using the supernatant of cattle faeces from three geographically distinct regions of the UK. There was no relationship between the concentrations of lactoferrin in faecal supernatant and serum, suggesting different metabolic sources of lactoferrin, or differences in the success of lactoferrin extraction from two different substrates, which requires further work to identify. Therefore, faecal lactoferrin may provide novel information that can provide new insight into animal health. Robust interpretation of faecal lactoferrin ELISA results will require substantial future work. Nevertheless, this successful proof-of-concept highlights how lactoferrin and potentially other immune-markers, can be quantified non-invasively.

## Supplemental Information

10.7717/peerj.8631/supp-1 Supplemental Information 1 Raw data that was collected and used within the analyses.

10.7717/peerj.8631/supp-2 Supplemental Information 2 Sheep and Deer results.

10.7717/peerj.8631/supp-3 Supplemental Information 3 Example of Lactoferrin Calibration Curve.

10.7717/peerj.8631/supp-4 Supplemental Information 4 Optical density of supernatant in relation to faecal dry matter. An additional mini-experiment conducted in tandem to the main experiment to test if the optical density of a supernatant (in it’s raw form, pre-ELISA) correlates to the concentration of lactoferrin as determined by the assays.

## Abbreviations

ANOVA: analysis of variance
EDTA: ethylenediaminetetraacetic acid
ELISA: enzyme-linked immunosorbent assay
GIN: gastrointestinal nematode(s)
H_2_SO_4_: sulfuric acid
HRP: horseradish peroxidase
OD: optical density
TBST: tris-buffered saline with Tween 20

## Additional Information and Declarations

### Field Study Permissions

The following information was supplied relating to field study approvals (i.e., approving body and any reference numbers): Permission for the cattle field sites was obtained by Andrew Cooke (Rothamsted Research) from the land owner and/or manager in each instance. No specific field permit was required. Permission for sheep and deer samples was obtained by the University of Edinburgh from the land owner and/or manager. No specific field permit was required. The single deer blood sample was taken with the landowner’s permission from an animal shot for food.

### Data Availability

The following information was supplied regarding data availability: Raw data is available as a Supplemental File.
