# Supplementary material for: Lactoferrin quantification in cattle faeces by ELISA
Source: PeerJ. 2020 Feb 27;8:e8631. doi: 10.7717/peerj.8631 (PMC7189889; doi:10.7717/peerj.8631)
Supplement: Supplemental Information 2 [file peerj-08-8631-s002.docx]

**Supplement X: Additional sheep and deer samples**

# X.1 Introduction

This supplement includes the data for the sheep and deer samples that were made available to the study. Cattle samples are also reported within this supplement for the convenience of the reader when making comparisons.

The structure of lactoferrin varies between species, however shares significant similarities. The more structurally similar that two forms of lactoferrin are, the more likely that an antibody, specialised to one form, will have a avidity to capture the other form. Shimazaki *et al.* (1991) [1] found that partial antigenic identity between goat and sheep lactoferrin and that the carbohydrate compositions of bovine, sheep, and goat lactoferrin were the same as each other, but different to that of humans. Similarities in lactoferrin across species raises the possibility that an ELISA designed to capture lactoferrin from one species may have an avidity to do so for lactoferrin from another.

# X.2 Method

All methodology was the same as in the main manuscript with the exception of any aspects described below.

## X.2.1 Sample populations

Faecal samples were collected from individuals from eight ruminant populations (three groups of cattle, two groups of sheep and two groups of deer), representing typical populations of farmed and wild ruminants in the UK (Table 1). Both sheep populations, both deer populations, and one of the cattle populations (S1, S2, D1, D2, C2 (Table X.1)) were grazing on pasture for their entire lifespan. Two of the cattle populations (C1, C3 (Table X.1)) were feeding on grass silage for at least one-month leading up to sampling. All domestic animals were considered healthy to the point where they had no known illness or infection worthy of intervention. Wild an extensive populations had no veterinary interventions during their lifetimes, no clear health concerns were present.

| Group | Species | Status | *n* faecals | *n* bloods |
| --- | --- | --- | --- | --- |
| C1 | Cattle | Domestic | 65 | 0 |
| C2 | Cattle | Domestic | 30 | 0 |
| C3 | Cattle | Domestic | 22 | 22 |
| S1 | Sheep | Wild | 88 | 7 |
| S2 | Sheep | Wild | 42 | 0 |
| D1 | Deer | Wild | 126 | 0 |
| D2 | Deer | Extensive | 0 | 1 |

Table X.1 - Sample numbers and associated information for ruminants used in this study. Where sample numbers exceed number of individuals, multiple samples were taken from the same individuals during different seasons. “Domestic” refers to animals kept in traditional farm settings, with regular human interaction, controlled grazing patterns, and fenced fields. “Extensive” refers to animals that were commercially reared but roamed freely within a large area with minimal management. Wild animals were feral, had no form of management intervention and roamed freely.

## X.2.1 Statistical analysis

Two-sample *T*-tests were used to determine if faecal supernatant ODs, for each species, were significantly above background levels (TBST blanks).

One-way ANOVA and a post-hoc Tukey test were used to assess differences in faecal lactoferrin concentrations between species. As there was only one deer serum sample, a 2-sample *T*-test was used to compare serum lactoferrin concentrations between cattle and sheep only.

# X.3 Results

## X.3.1 Controls, references, and calibration

Negative controls of just TBST were consistent across all plates and had a mean background optical density of 0.0486, ranging from 0.0467 to 0.0506, with a relative standard error of 0.77%. Reference material gave consistent curves with a mean relative standard error of 1.51% across all dilutions.

Based upon the results of three initial test plates, it was determined that a sample concentration of 50% was optimum for cattle and deer faecal assays, while 100% concentration was optimum for sheep faecal samples. At these dilutions, all samples yielded optical densities significantly above background levels, as determined using 2-sample *T*-tests that compared background levels to faecal supernatants of cattle (*T*_13, 115_ = 11.99, *p* < 0.0005), sheep (*T*_13, 130_= 4.72, *p* < 0.0005), and deer (*T*_13, 126_ = 8.80, *p* < 0.0005).

## X.3.2 Lactoferrin concentrations

A Grubbs’ test found one in the sheep serum data set (0.669 µg mL^-1^) which was excluded for all statistical analyses and graphing.

### X.3.2.1 Faecal samples

The mean lactoferrin concentration across all faecal samples (all species) was 0.091 µg mL^-1^ (S.E. 0.011) and 0.087 µg mL^-1^ (S.E. 0.020) in serum samples.

Cattle faecal samples yielded substantially higher lactoferrin concentrations compared to sheep and deer samples (means: 0.269, 0.008 and 0.013 µg mL^-1^ respectively). A One-way ANOVA found statistically significant differences in faecal lactoferrin concentrations between species (*F* = 77.50, *p* < 0.0005) with a post-hoc Tukey test confirming that cattle samples varied significantly from sheep and deer samples. Faecal lactoferrin concentrations from sheep and deer were not significantly different to each other (Figure 1).


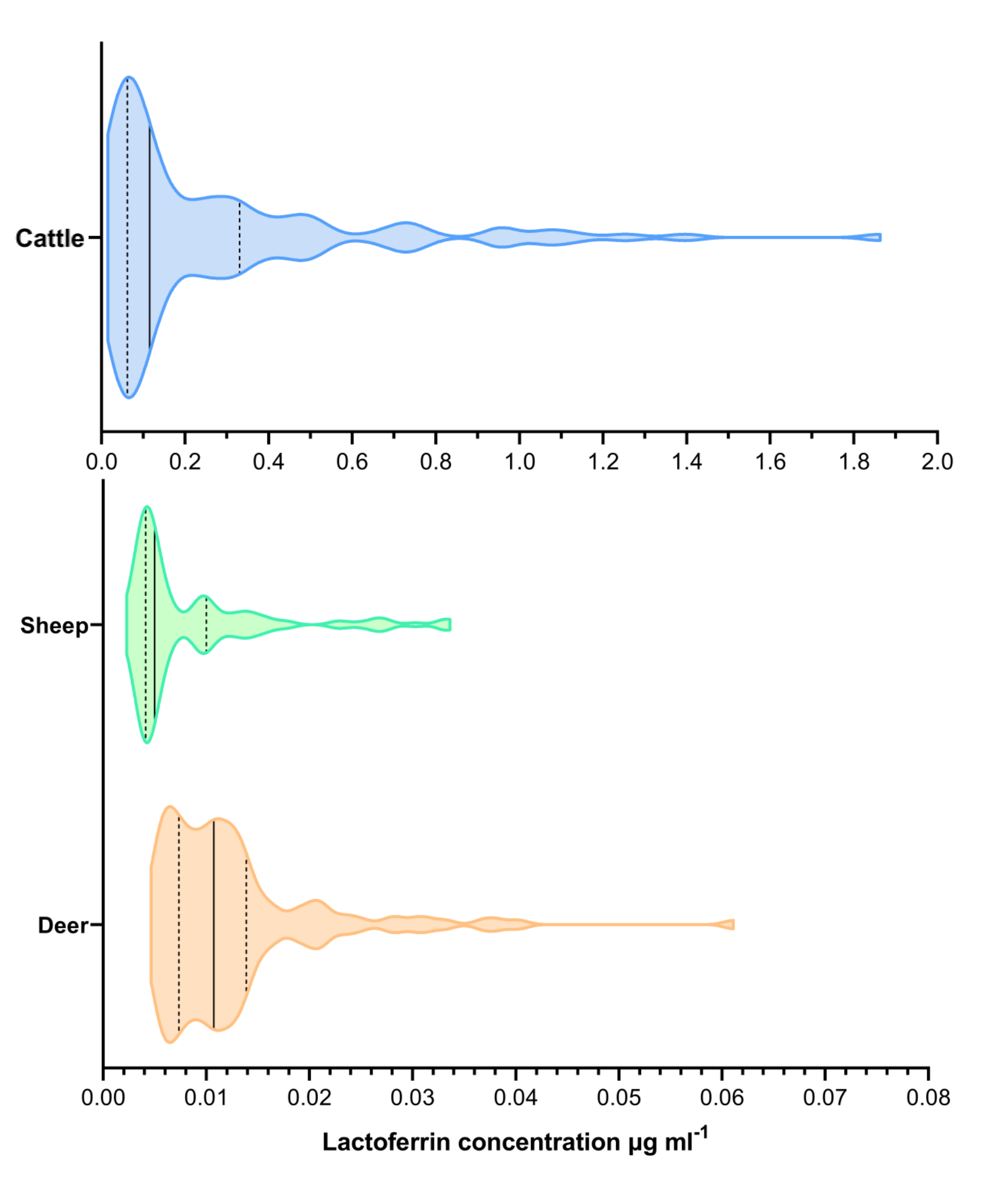


Figure X.1 – Violin plots showing the distribution of faecal lactoferrin concentrations (µg mL-1) of faecal samples from cattle (n = 115), sheep (n = 130), and deer (n = 126). The width of the plot represents the frequency Solid black lines represent median whilst dashed black lines represent quartiles. Note that x-axis differs for cattle and for sheep/deer, in which much lower levels of lactoferrin were measured

### X.3.2.2 Serum samples

The lactoferrin concentration in the single deer blood sample was 0.093 µg mL^-1^.

A 2-sample *T*-test showed no statistically significant difference between the serum lactoferrin concentrations derived from cattle or sheep (means: 0.074 and 0.036µg mL^-1^_,_ respectively) blood (*T*_22, 6_ = 2.40, *p* = 0.053) (Figure 2).


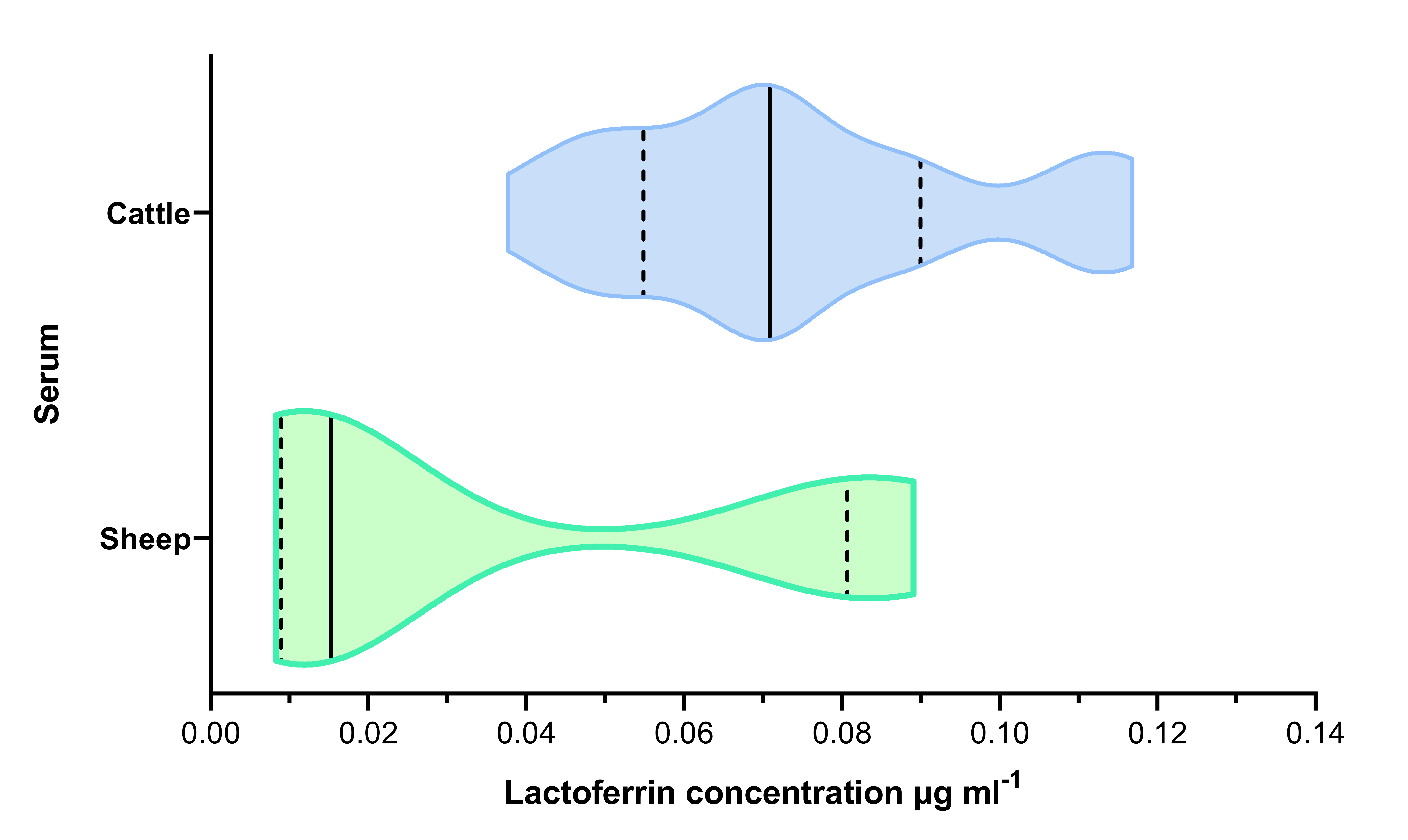


Figure 2 – Violin plot showing the distribution of serum lactoferrin concentrations (µg mL-1) from cattle (n=22) and sheep (n=6). The width of the plot represents the frequency. Solid black lines represent the median whilst the dashed black lines represent quartiles.

# X.4 Discussion

The sensitivity of a bovine ELISA to detect lactoferrin isotypes from non-bovine ruminants is unknown, although likely to be sub-optimal. In this study, we observed lower quantitites of lactoferrin in samples from sheep and deer compared with those from cattle and suggest that is likely a consequence of commercial optimisation of the assay for cattle using bovine reagents only. However, that issue can be accounted for, Dial *et al.* [2] successfully used antibodies to human lactoferrin for the analysis of bovine sample, using a western blot to verify reactivity and selectivity. Mass spectrometric analysis could also be performed to assess the aviditidy and performance of assays between sample species.

# X.5 References

1. Shimazaki K, Kawano N, Yoo YC. Comparison of bovine, sheep and goat milk lactoferrins in their electrophoretic behavior, conformation, immunochemical properties and lectin reactivity. Comp Biochem Physiol B. 1991;98:417–22.

2. Dial EJ, Hall LR, Serna H, Romero JJ, Fox JG, Lichtenberger LM. Antibiotic Properties of Bovine Lactoferrin on Helicobacter pylori. Dig Dis Sci. 1998;43:2750–6.
