# Supplementary material for: Lactoferrin quantification in cattle faeces by ELISA
Source: PeerJ. 2020 Feb 27;8:e8631. doi: 10.7717/peerj.8631 (PMC7189889; doi:10.7717/peerj.8631)
Supplement: Supplemental Information 3 [file peerj-08-8631-s003.docx]

**Supplement Y – Lactoferrin calibration curve example**

Reference material underwent the ELISA protocol at seven concentrations along with TBST blanks. A sample of reference material of 0.500 µg mL^-1^ was used and serially diluted, halving concentration each time, until a final concentration of 0.0078125 µg mL^-1^.

The below example is the reference material calibration curve from Plate #1 (Figure Y.1). Data for all reference curves, including optical densities of reference material and curve equations, are available in the data supplement.


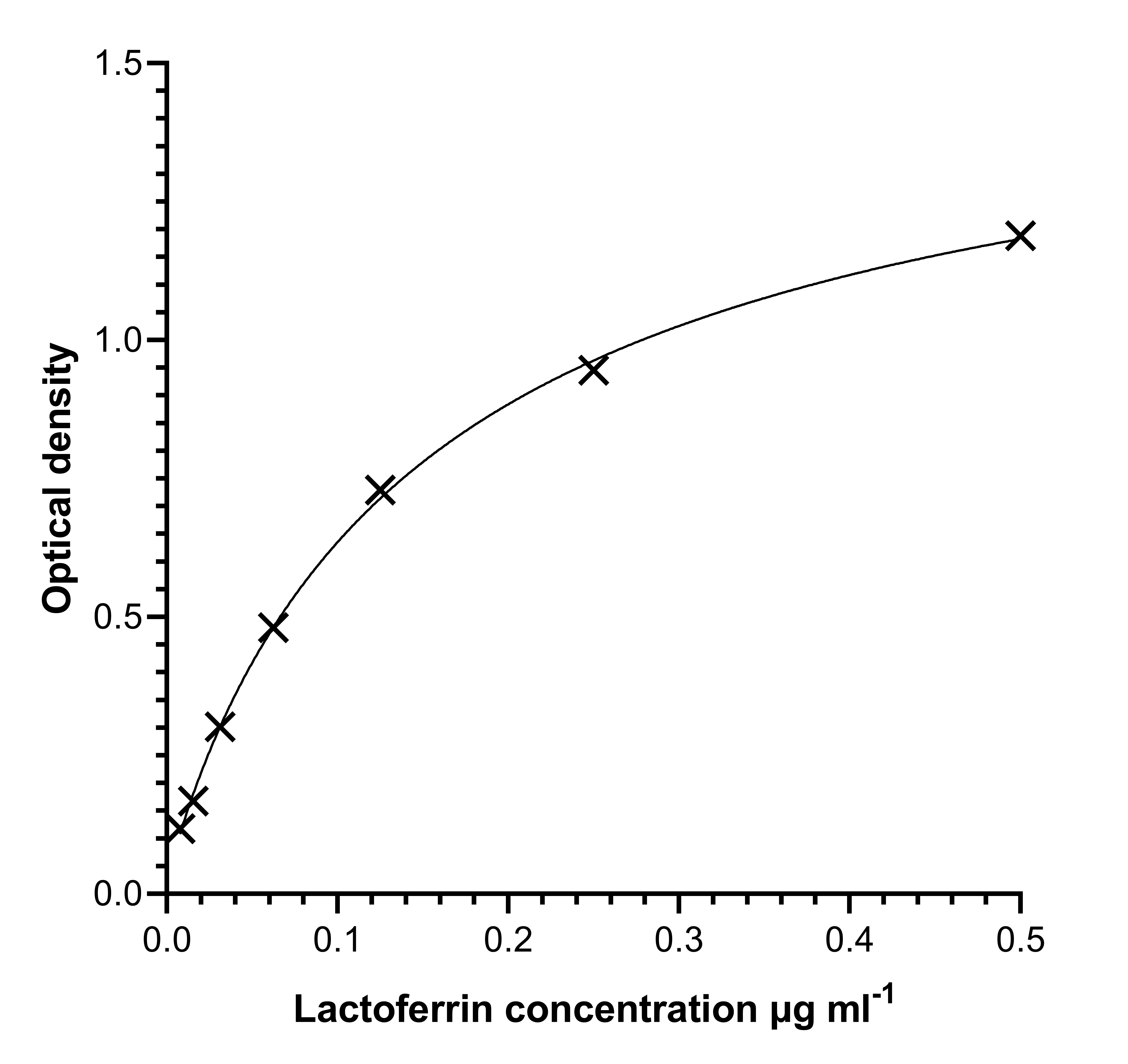


Figure Y.1 - Reference material calibration curve generated for Plate #1.
