# Supplementary material for: Lactoferrin quantification in cattle faeces by ELISA
Source: PeerJ. 2020 Feb 27;8:e8631. doi: 10.7717/peerj.8631 (PMC7189889; doi:10.7717/peerj.8631)
Supplement: Supplemental Information 4 — An additional mini-experiment conducted in tandem to the main experiment to test if the optical density of a supernatant (in it’s raw form, pre-ELISA) correlates to the concentration of lactoferrin as determined by the assays. [file peerj-08-8631-s004.docx]

**Supplement Z: Supernatant optical density and dry matter**

**Z.1 Introduction**

The moisture content of fresh faeces depends on animal diet and state of hydration. Temporal variation in faecal moisture content may significantly influence the concentrations of components, including lactoferrin, within the faeces. Thus, a simple test was conducted to investigate whether faecal moisture content or sample background optical density were related to lactoferrin concentrations.

**Z.2 Method**

For 56 of the cattle faecal samples (from groups C1, C2, and C3), faecal supernatants at 1:2 (w:v) ratio of faeces to protease inhibitor, were measured to determine optical density of the sample solution (as opposed to of the enzyme – substrate interaction within the ELISA methodology). This was conducted twice using 100 µL and 50 µL of supernatant. Blank 96-well plates were initially read to determine background optical density. Aliquots of each supernatant were pipetted into individual wells, avoiding the outer two rows and columns, to avoid potential edge effects. Plates were then read using a plate reader, to determine optical density, from which the background value was subtracted. Supernatant optical densities were then correlated to lactoferrin concentration and subsequent regression analysis conducted.

Twenty-nine of the faecal cattle samples (from groups C1, C2, and C3) were analysed for dry matter content by gravimetric loss at 65°C to a constant weight.

**Z.3 Results**

Optical density of faecal supernatants correlated significantly with lactoferrin concentration at 100 µL (*ρ* = 0.377, *p* = 0.004) but not 50 µL (Pearson’s correlation = 0.135, *p* = 0.135) (Figure Z.1). Subsequent regression analysis of lactoferrin concentration as a response to optical density at 100 µL yielded an r^2^ of 14.2%.


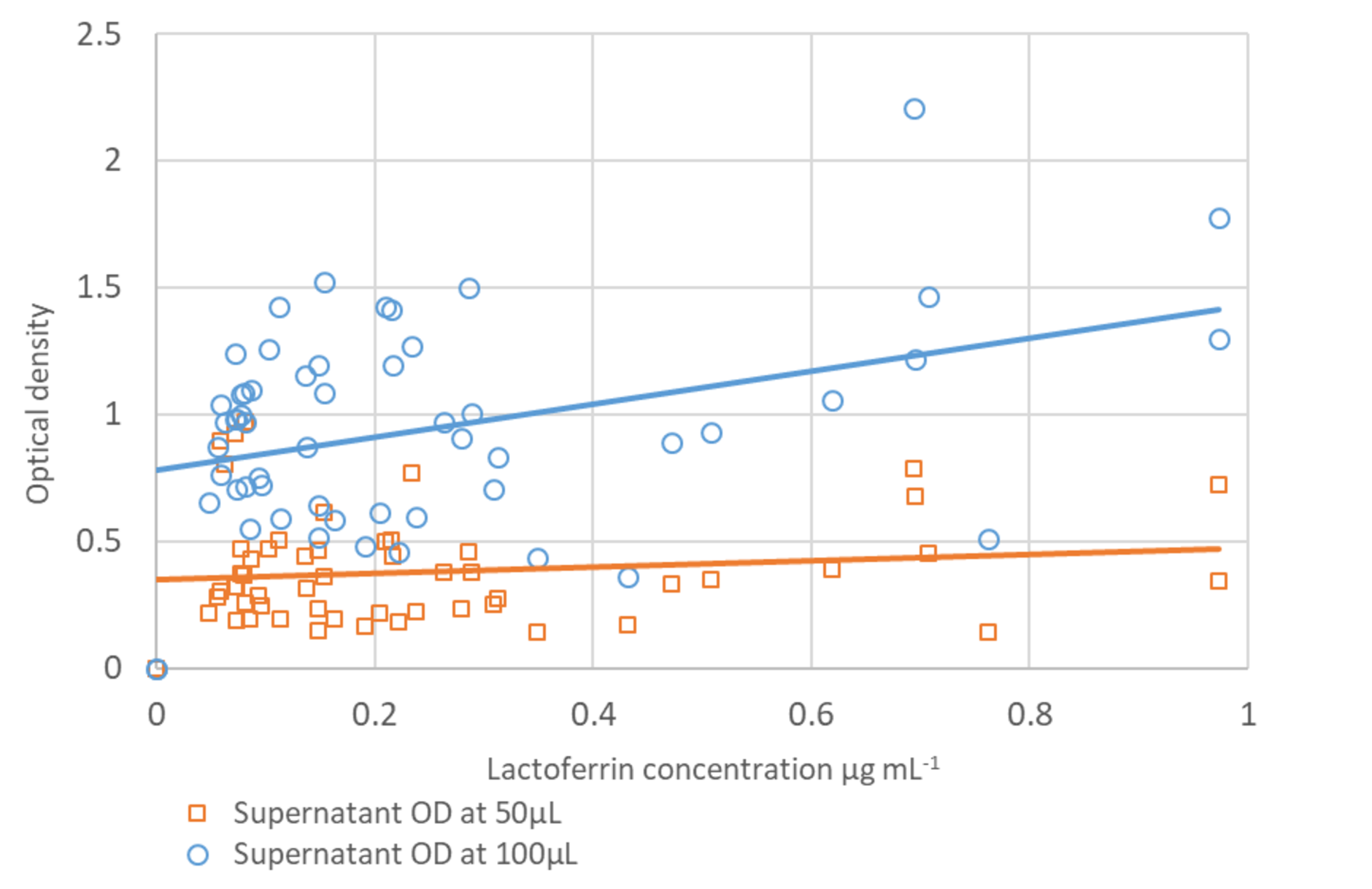


Figure Z.1 - Scatterplot with trendlines showing the relationship between faecal supernatant optical densities (at 50 µL and 100 µL) and lactoferrin concentration (µg mL-1).

Dry matter content of faeces was not significantly correlated with lactoferrin concentration (*ρ* = -0.148, *p* = 0.161) (Figure Z.2).


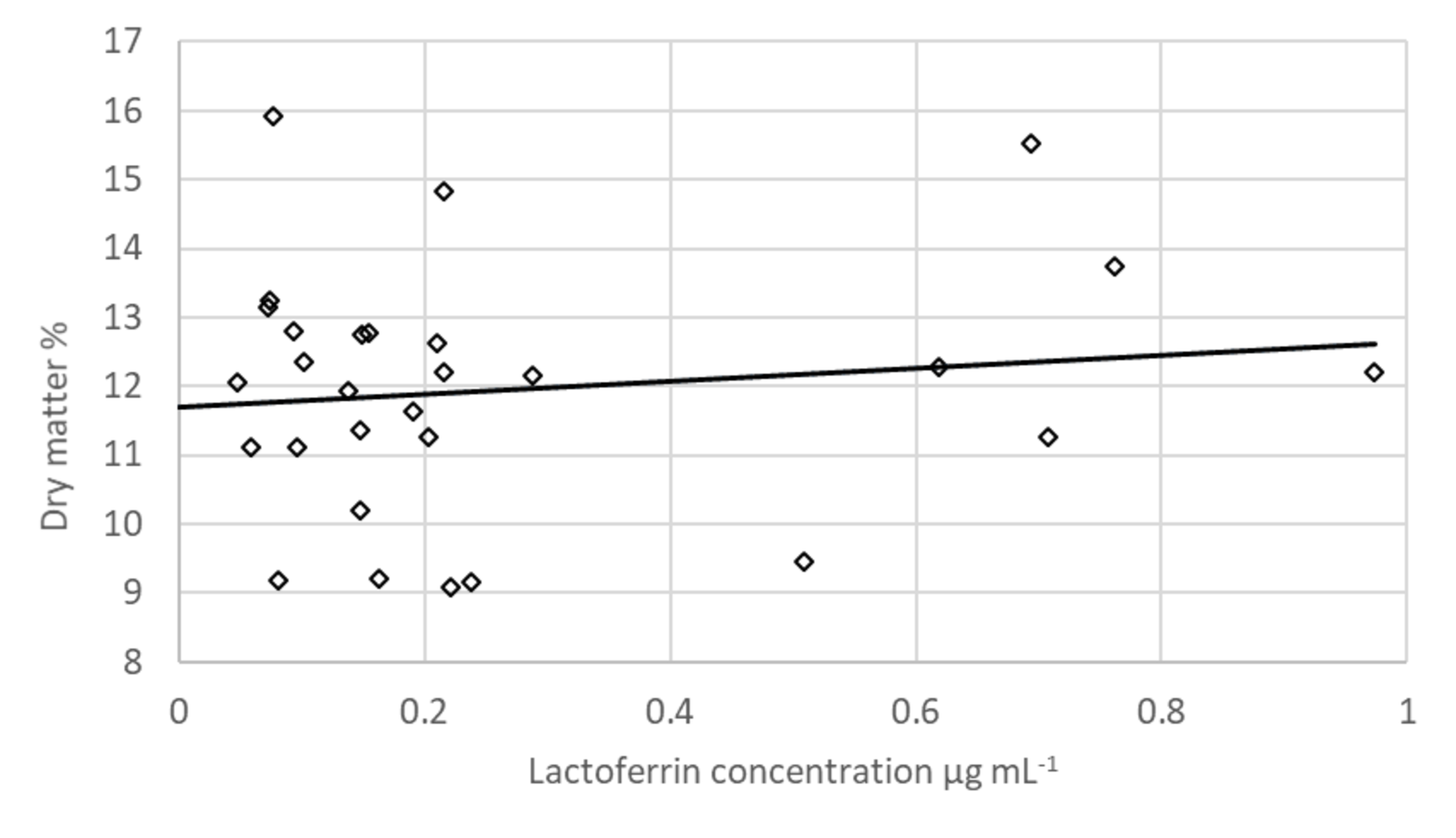


Figure Z.2 - Scatterplot with trendlines showing the relationship between faecal dry matter content (%) and faecal lactoferrin concentration (µg mL-1).

**Z.4 Discussion**

The comparison of faecal sample dry matter and background optical density to lactoferrin concentration highlighted an important consideration when using faecal material for any molecular quantification techniques. However, correlations were relatively weak and the reasoning for why samples correlated at 100 µl but not 50 µl is not immediately clear. It may simply be a case that 50 µl does not provide a great enough volume and mass for sufficient absorbance. The physical composition of faeces can vary greatly [38], not just between individuals, but for the same individual at different times, diluting or concentrating immunomarkers within sampled faeces. Furthermore, diarrhoea, which generates a high faecal moisture content, can be symptomatic of gastrointestinal disease, pathology, or infection [39–41], creating a confounding factor. The mechanism driving the significant relationship between supernatant optical density and lactoferrin is unknown but may relate to the effects of gut damage on faecal composition or the concentration of faeces itself in relation to water content. Further investigation is required to determine any systematic adjustment or interpretation of ELISA results related to potential confounding factors.
